# Association Between a Prognostic Gene Signature and Functional Gene Sets

**DOI:** 10.4137/bbi.s1018

**Published:** 2008-09-22

**Authors:** Manuela Hummel, Klaus H. Metzeler, Christian Buske, Stefan K. Bohlander, Ulrich Mansmann

**Affiliations:** 1 Department of Medical Informatics, Biometry and Epidemiology, University of Munich, Germany; 2 Bioinformatics and Genomics Program, Centre for Genomic Regulation (CRG), Barcelona, Spain; 3 Laboratory of Leukemia Diagnostics, Department of Internal Medicine III, University Hospital Großhadern, University of Munich, Germany; 4 Department of Statistics, University of Munich, Germany

## Abstract

**Background:**

The development of expression-based gene signatures for predicting prognosis or class membership is a popular and challenging task. Besides their stringent validation, signatures need a functional interpretation and must be placed in a biological context. Popular tools such as Gene Set Enrichment have drawbacks because they are restricted to annotated genes and are unable to capture the information hidden in the signature’s non-annotated genes.

**Methodology:**

We propose concepts to relate a signature with functional gene sets like pathways or Gene Ontology categories. The connection between single signature genes and a specific pathway is explored by hierarchical variable selection and gene association networks. The risk score derived from an individual patient’s signature is related to expression patterns of pathways and Gene Ontology categories. Global tests are useful for these tasks, and they adjust for other factors. GlobalAncova is used to explore the effect on gene expression in specific functional groups from the interaction of the score and selected mutations in the patient’s genome.

**Results:**

We apply the proposed methods to an expression data set and a corresponding gene signature for predicting survival in Acute Myeloid Leukemia (AML). The example demonstrates strong relations between the signature and cancer-related pathways. The signature-based risk score was found to be associated with development-related biological processes.

**Conclusions:**

Many authors interpret the functional aspects of a gene signature by linking signature genes to pathways or relevant functional gene groups. The method of gene set enrichment is preferred to annotating signature genes to specific Gene Ontology categories. The strategies proposed in this paper go beyond the restriction of annotation and deepen the insights into the biological mechanisms reflected in the information given by a signature.

## Introduction

Gene signatures are sets of genes along with an algorithm. Combined, the two allow for the discrimination between different biological entities (Kostka and Spang, 2008). While the focus of signatures is on discrimination, the functional aspect causing different phenotypes is hidden and not directly accessible from the signature’s information. This paper will introduce computational strategies for elucidating the functional context behind gene signatures.

In patient-oriented medicine it is of great importance to accurately classify patients into homogeneous diagnostic or prognostic subgroups and to supply therapies targeted to those subgroups (Schlenk et al. 2008). Gene signatures derived by microarray technology play an important role in refining or supplementing existing diagnostic and prognostic factors such as staging systems (van de Vijver, 2002). But even if a signature is validated, proven to be accurate, and generalizable, there is still a long way to go before it is part of medical decision making. Wyatt and Altman (Wyatt and Altman, 1995) describe several features of prognostic models that facilitate their application in medical routines. A critical issue is whether the diagnostic/prognostic model can be interpreted in terms of the disease process.

In general, the biological interpretation of a gene signature follows a simple strategy: authors search for articles in which signature genes were described as relevant to the biological process under study. Interpreting different signatures that are developed for the same specific purpose from different research groups this way is impaired by the fact that the corresponding gene lists barely overlap. An example from colon cancer is instructive: three prognostic signatures show one single gene as common (Wang et al. 2004), (Barrier et al. 2006), and (Lin et al. 2007). The signatures seem to differ even if the same clinical population and the same endpoint are considered. Yu et al. (Yu et al. 2007) studied a similar example in breast cancer and found that the divergent gene signatures represent similar biological processes. An example from leukemia was discussed by Radmacher et al. (Radmacher et al. 2006). But discordance between signatures does not automatically imply deficiencies in the derivation or differences with respect to their biological content.

Yu et al. (Yu et al. 2007) used the enrichment of signature genes in the Gene Ontology (GO) (Ashburner et al. 2000) to identify the functional implications of the signature. The association between the expression profile of enriched gene sets and the patient prognosis was verified using globaltest (Goeman et al. 2004). The authors even proposed the development of predictive signatures on the basis of gene sets that were shown to play an important role in the biology of the disease under study. This idea has also been discussed by Lottaz and Spang (Lottaz and Spang, 2005) as well as by Tai and Pan (Tai and Pan, 2007). Classification strategies using annotated genes are dependent on the state of functional gene annotation and exclude a large part of information on gene expression that is available in signature genes that have not yet been integrated into a functional context. We aim for a signature interpretation that does not exclude signature genes not yet annotated.

We are interested in gene regulatory mechanisms that are affected by the signature. The correlation between the expression patterns of the signature and KEGG pathways (Kyoto Encyclopedia of Genes and Genomes, http://www.genome.jp/kegg/(Kanehisa et al. 2006)) is explored by the GlobalAncova approach (Mansmann and Meister, 2005; Hummel et al. 2008). The signature genes with the most influence on the pathway’s expression profile are identified by a hierarchical variable selection procedure (Meinshausen, 2008). Furthermore, we propose to study a gene association network (Schäfer and Strimmer, 2005) for the combined set of signature and pathway genes.

We illustrate the proposed strategies with a dataset of 163 patients suffering from acute myeloid leukemia (AML) (Metzeler et al. 2008). The dataset will be publicly available following publication of the original paper. We have developed a signature and a prognostic score to predict patient survival. Besides analyzing the role of individual signature genes, we use the prognostic score as a surrogate for the whole signature. The association between the prognostic score and relevant pathways or Gene Ontology categories (Ashburner et al. 2000) is analyzed by GlobalAncova (Mansmann and Meister, 2005; Hummel et al. 2008) and globaltest (Goeman et al. 2004). Gene Ontology categories are scored by the focus level method (Goeman and Mansmann, 2008), which corrects for multiple testing while accounting for the special hierarchical structure of the GO. We also discuss the interaction between the signature and other relevant factors such as mutations.

## Materials and Methods

**Figure 1:**
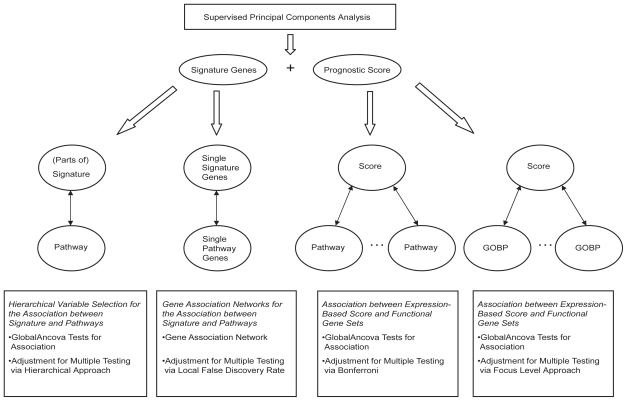
Schematic diagram of the analyses.

The signature for predicting survival in AML was developed using the Supervised Principal Components approach which selects genes whose expression patterns are correlated with survival and, based on those, generates a prognostic score (Bair and Tibshirani, 2004). Details about the signature development can be found in (Metzeler et al. 2008). Figure 1 gives an overview over the analyses described in the following.

### Hierarchical variable selection for the association between signature and pathways

The correlation between the expression profiles of the signature and the pathway (*co-expression*) is explored with GlobalAncova (Mansmann and Meister, 2005; Hummel et al. 2008). GlobalAncova is based on a flexible individual linear model for each gene of a specific set and summarizes the relevance of a regression variable in order to explain the gene expression in the given set. A single signature gene or a subgroup of genes can serve as the dependent variable(s) of interest. The goal is to identify the signature genes that affect the global expression of a specific pathway. This is a variable selection problem. To avoid a large number of false positive selections (Efron et al. 2004), Meinshausen (Meinshausen, 2008) proposes a strategy that controls the errors in variable selection. The procedure renders a set of relevant signature genes that contains false positives with probability α (for a predefined error level α). Meinshausen’s hierarchical variable selection subsequently tests clusters of similar genes while keeping control of the family-wise error rate (FWER).

To this end, the signature is divided into nested groups via hierarchical clustering with a correlation-based distance measure. The root of the resulting binary tree corresponds to the whole signature, and its leaves are the single genes. Starting from the root, each cluster of signature genes is used as the set of regressors for the GlobalAncova test. Meinshausen adjusts each test for multiple testing adapted to the size of the respective cluster. Tests with large clusters of dependent variables are adjusted only weakly; whereas, single genes receive a Bonferroni-type adjustment. The algorithm proceeds as long as there are significant test results until, in the end, the influences of single signature genes or small groups of genes on the expression profile within the pathway are tested. We use a conservative error rate of α = 0.01.

### Gene association networks for the association between signature and pathways

Alternatively, one may focus on gene-by-gene interactions in order to see which signature gene is related to which pathway gene. For this, we estimate gene dependency networks as proposed in Schäfer and Strimmer (Schäfer and Strimmer, 2005). The resulting graphical model represents partial correlations, i.e. the correlations between two genes, while controlling for all the remaining genes. In order to avoid false positive edges in the network, an edge is only drawn if the respective genes have a partial correlation whose local false discovery rate (fdr) is smaller than 0.2. The networks are estimated using the R package GeneNet (Opgen-Rhein et al. 2006).

### Association between expression-based score and functional gene sets

GlobalAncova (Mansmann and Meister, 2005; Hummel et al. 2008) and globaltest (Goeman et al. 2004) are used to test the association between the microarray-based prognostic score and the expression profile of a gene set while adjusting for other risk factors. GlobalAncova is also able to study the interaction between the prognostic score and a second risk factor (for example, a mutation). This enables researchers to determine whether the expression profile within a pathway is regulated by the prognostic score differently for patients with versus without the specific mutation.

The proposed models are applied to pathways and Gene Ontology Biological Process (GOBP) categories (Ashburner et al. 2000). Since the GOBP consists of several thousand terms, an adjustment for multiple testing is required. We choose the focus level method (Goeman and Mansmann, 2008) that controls the FWER and accounts for the special hierarchical structure of the GO graph. The procedure is available in the Bioconductor packages globaltest and GlobalAncova. Pathway analysis is corrected by the Bonferroni method (Dudoit et al. 2003).

All analyses are done with the open source software R (The R Development Core Team 2007) (version 2.6.0) and Bioconductor (Gentleman et al. 2004) (version 2.1).

## Data

In order to illustrate this methods, we will discuss a data set of 163 adult AML patients with normal karyotype. Clinical data and Affymetrix HG-U133 A + B microarrays are available for all patients. The mutation status of two prognostically relevant genes is also available: *NPM1* (nucleophosmin 1, Entrez GeneID 4869) and *FLT3* (fms-like tyrosine kinase 3, Entrez GeneID 2322) (Marcucci et al. 2005). Details on the development and validation of the signature and the corresponding prognostic score can be found in (Metzeler et al. 2008).

The 44,754 measured Affymetrix probesets map to 18,481 unique gene symbols. All analyses are applied on a gene-basis instead of a probeset-basis. For each gene that maps to more than one probeset, one probeset is chosen to represent the gene’s expression value. We choose the probesets at random since there is no “optimal” way for collapsing probesets to genes. Moreover, the probesets within the AML signature corresponding to the same gene, respectively, show high correlations.

We considered KEGG pathways (Kanehisa et al. 2006), which generally represent known gene interaction networks. We use the information provided by the KEGG database about the association of genes to pathways for defining our pathway gene sets. The information about interactions between genes within the pathways is not used in our approaches. Fifteen genes of the 67 signature genes are annotated to KEGG pathways. Thirty-nine signature genes are annotated to terms of the Gene Ontology Biological Processes (Ashburner et al. 2000).

## Results

### Hierarchical variable selection for the association between signature and pathways

**Figure 2:**
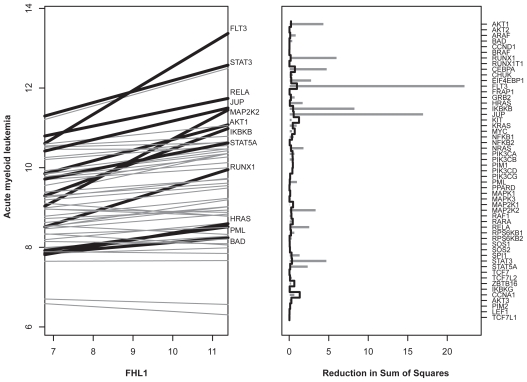
Relation between signature gene FHL1 and AML pathway. Left: Linear effects of signature gene FHL1 on the single genes within pathway ‘acute myeloid leukemia’ (hsa05221). Significant gene-wise positive effects are indicated by thick black lines and corresponding gene names. Right: Corresponding GlobalAncova gene plot. The bar height indicates gene-wise contributions to the GlobalAncova statistic. Bar height and reference line correspond to the numerator and denominator of gene-wise F-statistics.

First, we wanted to know which signature genes influence the pathway for Acute Myeloid Leukemia (AML) (KEGG identifier hsa05221). The AML oncogene *RUNX1* (Entrez GeneID 861) belongs to both the signature and the AML pathway. The specific analysis for the gene *FHL1* (known to be associated with a poor response to chemotherapy (Heuser et al. 2005), Entrez GeneID 2273) uses GlobalAncova. The expression of the *FHL1* gene influences the expression profile of the AML pathway (p < 0.0001). Figure 2 illustrates the estimated linear effects of *FHL1* on the pathway genes, as calculated on a gene-wise basis. Several effects stay significant after applying Bonferroni correction. The right side of Figure 2 is the GlobalAncova gene plot that shows gene-wise contributions to the global test statistic. Both plots support the strong correlation between *FHL1* and the pathway.

**Figure 3:**
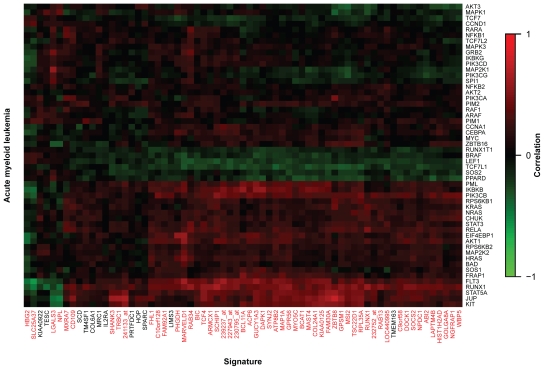
Heatmap of gene-wise correlations between signature genes and single genes of the AML pathway. Signature genes correspond to columns; genes within the pathway ‘acute myeloid leukemia’ (hsa05221) correspond to rows. Red spots indicate large positive correlation, green spots indicate large negative correlation, and dark spots stand for correlations around zero. Labels of signature genes are given in red for those genes that show a significant influence on the AML pathway according to the hierarchical variable selection procedure. Affymetrix probeset identifiers are used when no annotation to gene symbols exist.

The correlation between individual signature and pathway genes is shown in Figure 3. Most of the signature genes (55 of 67) are strongly associated with the expression pattern of the AML pathway. The results show the influence on a specific pathway from genes not annotated to any pathway and genes annotated to other pathways. Most of the signature genes are positively correlated with important oncogenes in AML such as *FLT3* or c-*KIT* (Entrez GeneID 3815).

**Figure 4:**
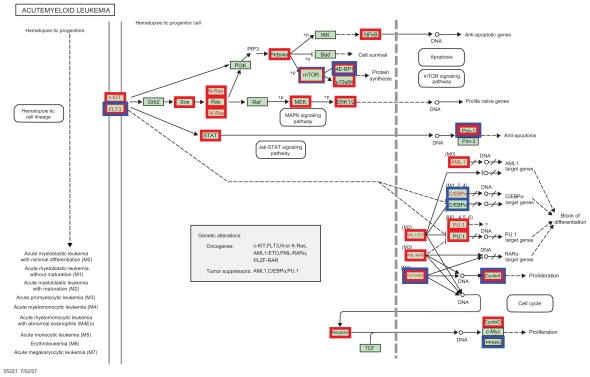
KEGG pathway ‘acute myeloid leukemia’ (hsa05221). Red boxes mark involved genes that correlate significantly with at least one of the signature genes. Blue boxes mark genes that show a significant partial correlation (in the gene association network) to at least one of the signature genes.

**Figure 5:**
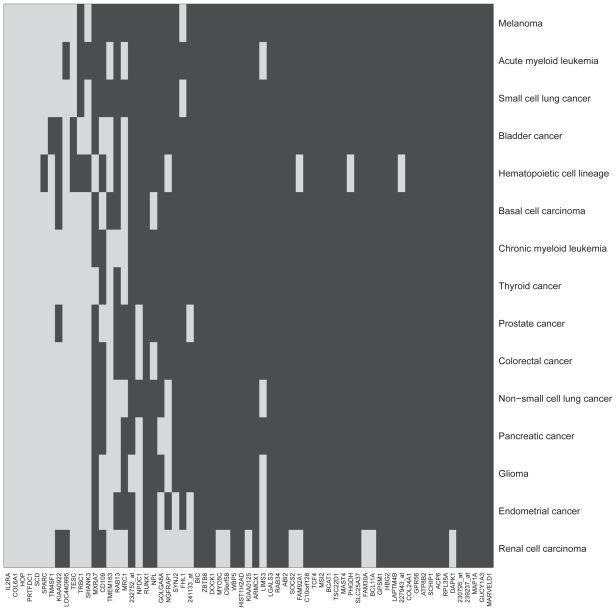
Result of hierarchical variable selection for 15 cancer-specific KEGG pathways. Rows indicate pathways; columns show the 67 signature genes. Squares are dark gray rather than light gray if there is a significant influence of the respective signature gene on the respective pathway (adjusted p-value = 0.0067).

A more sophisticated correlation structure is presented in Figure 4, where genes in the AML pathway (original graph taken from http://www.genome.jp/dbget-bin/www_bget?pathway+hsa05221) are marked with a red box when they correlate significantly with signature genes (Bonferroni corrected for multiplicity). There is a dense interaction between the AML pathway and signature. A systematic search for signature genes that are co-regulated with genes in the acute myeloid pathway uses the hierarchical variable selection procedure of Meinshausen (Meinshausen, 2008). A similar analysis can be performed for all (200) KEGG pathways available in the corresponding R annotation package that contain at least one gene represented on the Affymetrix HG-U133 A + B genechip. Figure 5 shows which signature genes influence gene expression in 15 cancer-specific pathways. In Supplementary Figure 1 results are shown for the remaining 185 pathways.

Each signature gene is correlated with at least one of the pathways. On average, a gene is co-regulated with 122 of the 200 pathways. Only three pathways do not relate to any signature gene (‘Phenylpropanoid biosynthesis’ (hsa00940) ‘Methane metabolism’ (hsa00680), ‘D-Arginine and D-ornithine metabolism’ (hsa00472)). On average, a pathway was influenced by 41 of the 67 signature genes.

### Gene association networks for the association between signature and pathways

**Figure 6:**
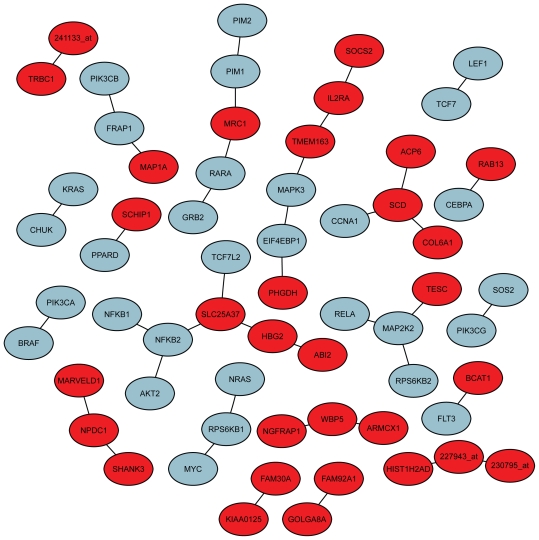
A gene association network that shows relations between signature and AML pathway genes. Blue nodes correspond to genes in the pathway ‘acute myeloid leukemia’ (hsa05221), and red nodes correspond to genes in the prognostic signature. Only edges corresponding to partial correlations considerably different from 0 (with fdr adjusted p-values < 0.2) are displayed. Correspondingly, only genes that are connected by an edge to another gene are shown. Affymetrix probeset identifiers are used when no annotation to gene symbols exist.

A gene association network (Schäfer and Strimmer, 2005) was estimated for the combined set of signature and ‘acute myeloid leukemia’ pathway genes. Figure 6 displays the observed connections and hides genes that were not connected. In the KEGG pathway graph (Fig. 4), the blue boxes highlight the elements that show a connection to the signature, according to the gene association network.

### Association between expression-based score and functional gene sets

We look for gene groups whose expression profile is related to the prognostic score. Additionally, we want to control for the influence of the *FLT3* mutation status. Two hundred KEGG pathways and 4,779 GOBP terms were scored. GlobalAncova and globaltest were used to establish the influence of the prognostic score adjusted for *FLT3* mutation status. The FWER was controlled by the Bonferroni method in the pathway analysis and by the focus level procedure (Goeman and Mansmann, 2008) in the GO analysis.

**Figure 7:**
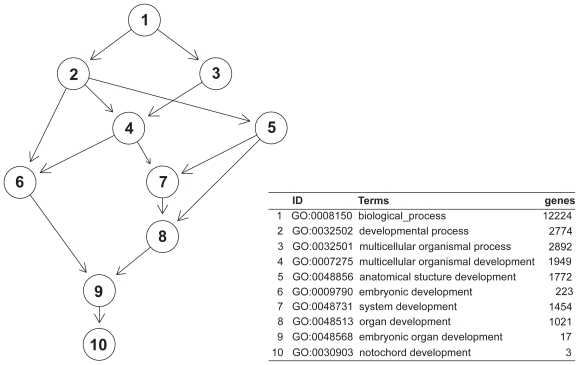
Result from GO scoring with the focus level procedure. GOBP terms are shown whose expression patterns are significantly affected by the interaction between expression-based prognostic score and *FLT3* mutation status.

**Figure 8:**
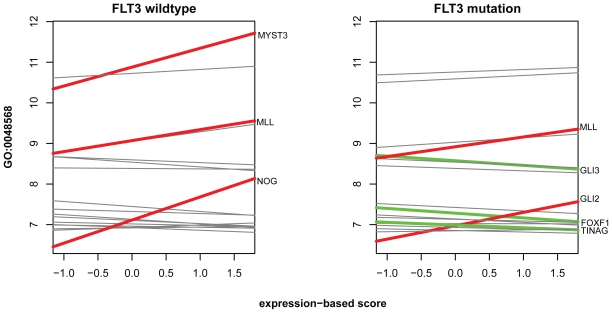
Gene-wise linear effects of the expression-based prognostic score on GOBP term ‘GO:0048568: embryonic organ development’. The term corresponds to node 9 in Figure 7. Significant (after simple Bonferroni correction) effects are indicated by red lines for positive effects and green lines for negative effects. The corresponding gene names are shown. Effects are calculated separately for patients with (right) and without (left) mutation in *FLT3*.

The results are presented in Table 1. From the 200 pathways, GlobalAncova detected 168 pathways at the significance level of 1% and globaltest detected 167, with an overlap of 166 pathways. In the GO analysis we detected 1781 (GlobalAncova) and 1751 (globaltest) terms with an overlap of 1730, also with a level of 1%. We were interested in functional groups in which the effect of the prognostic score is different between the groups defined by FLT3 mutation status. The pathway analysis did not yield any significant finding. When testing all GOBP terms for an interaction effect, a subgraph of 10 terms was detected (Fig. 7). The most specific terms are ‘notochord development’ (GO:0030903) and ‘embryonic organ development’ (GO:0048568). Table 2 shows the genes annotated to GO:0048568. Figure 8 shows the differential co-regulation between the prognostic score and individual genes within this GO term.

## Discussion

We provide strategies to elucidate the biological context of gene signatures. Though the methodologies used are not new, we apply them in a novel context. The work was motivated by the observation that a large part of the signature genes are not annotated to pathways or Gene Ontology terms. This excludes large parts of the signature from Gene Set Enrichment analyses and restricts the elucidation of a signature’s cellular context.

Besides a focused analysis for the single components of a signature, we also study co-regulation of the prognostic score with pathways or Gene Ontology Biological Process categories. The prognostic score offers a ‘summary’ of the relevant gene expression information included in all the signature genes.

The methods are illustrated on a prognostic signature for AML patients with normal karyotype. We propose the exploration of the interaction in expression profiles between a signature and functional pathways of interest by either testing linear relations with GlobalAncova (Mansmann and Meister, 2005; Hummel et al. 2008) or globaltest (Goeman et al. 2004), or by estimating gene association networks (Schäfer and Strimmer, 2005).

The idea of relating signatures with functional gene sets was already proposed by Yu et al. (Yu et al. 2007). The authors used gene set enrichment analysis in order to detect gene sets that contain (relatively) large numbers of signature genes. The concept provided in this paper is different from the concept of Yu et al. (Yu et al. 2007). Gene set enrichment detects biological processes that best *represent* the signature; whereas, we try to find gene sets that *interact* with it. Gene set enrichment concentrates on gene names and ignores the observed expression values. It also studies the randomness of membership in groups (is there an over-representation of statisticians in our institute compared to the statisticians working within the medical college?). Gene set enrichment does not answer questions such as whether the activity in one group is relevant to the activities observed in another group.

Furthermore, gene set enrichment restricts the analysis to the annotated genes of a signature. Our approach extends the analysis and allows the study of relationships between sets of not yet annotated genes and annotated gene groups. Only 15 genes of the signature are annotated to KEGG pathways. Thirty-nine of the 67 signature genes cannot be annotated to terms of the GO Biological Processes.

The KEGG pathway ‘acute myeloid leukemia’ is used as a specific example. Though only one gene in the signature (*RUNX1*) belongs to this pathway, many other signature genes show a strong correlation to the expression of several genes within the pathway. In particular, large parts of the signature correlate with well-known AML oncogenes such as *FLT3* or c-*Kit*. Some of the signature genes related to the AML pathway by the GlobalAncova-based approach and by the gene association networks are reported to play a role in other types of tumors, e.g. *BCAT1* (Entrez GeneID 586) (Rodríguez et al. 2003) and *SCHIP1* (Entrez GeneID 29970) (Scoles, 2008).

The activity of 55 of the 67 signature genes can be associated (hierarchical variable selection) to the AML pathway. Twenty-four of the 55 genes are not annotated to another KEGG pathway. *SHIP1* is one of these 24 genes.

The results of the hierarchical variable selection are not directly comparable to the results given by the graphical model of the interaction networks. First, the graphical model exhibits fewer associations between signature and pathway. Second, there were edges present in the estimated network; whereas, the corresponding signature genes did not show a significant influence on the AML pathway in the hierarchical approach.

Those differences do not have straightforward explanations. They are due to the different concepts of Pearson and partial correlation. A pair of genes, A and B, can have a detectable partial correlation, while the correlation of A or B to some other genes may be stronger than the correlation between A and B themselves. Within the AML pathway graph provided by KEGG (Fig. 4), the blue boxes highlight the parts that correspond to interactions between signature and pathway as given by the gene association network in Figure 6. There is an overlap between the red and blue boxes in Figure 4. So despite their differences, the hierarchical approach and the graphical model do show some convergence. The latter approach led to a sparser connectivity between signature and pathway.

We use pathways as lists of genes in our approach. The form and direction of interrelationships are not captured by our methods. Furthermore, the relationships depicted in the KEGG representation are of a different nature than those estimated by gene interaction networks. For example, the connections between genes within the AML pathway detected by the graphical model (Fig. 6) do no correspond directly to the connections known from laboratory research (Fig. 4).

The comparability of results from GlobalAncova and globaltest has been discussed in the literature (Mansmann and Meister, 2005; Liu et al. 2007). From the large amount of significant findings we concluded that patients with different score values were quite variable with respect to many kinds of biological processes and pathways. This underlines the relevance of the signature and the corresponding score.

The analysis also reveals the interaction of the *FLT3* status of a patient and the prognostic score in specific functional gene groups. The GOBP group GO:0048568 ‘embryonic organ development’ shows that the expression profiles in wild-type patients are influenced in a different way than for patients with the *FLT3* mutation.

The integration of gene signatures into a biological (functional, cellular) context is the starting point for basic research in molecular medicine, in order to elucidate the relationship between diagnostic or prognostic differences and differences in the disease process. The methods presented in this paper aim to broaden the scope of available strategies. They map out information that is helpful in guiding the design of new experiments on model organisms. They also produce information that is useful for improving annotation activities.

## Supplementary Material

Figure S1 Result of hierarchical variable selection for 185 (non-cancer-specific) KEGG pathways. Rows indicate pathways; columns show the 67 signature genes. Squares are dark gray rather than light gray if there is a significant influence of that signature gene on that pathway (adjusted p-value = 0.0067). A legend with pathway IDs and names is given in Supplementary Table 1.

Table S1 Legend for Supplementary Figure S1.

## Figures and Tables

**Table 1:** Result of pathway and GO analysis when testing the prognostic score adjusted by *FLT3* status. The numbers of significant findings obtained by GlobalAncova and globaltest after FWER correction at a level of 1% are shown. Additionally, the numbers of gene sets found by both global test approaches and total numbers of gene sets are given.

	KEGG	GOBP
GlobalAncova	168	1781
globaltest	167	1751
Overlap	166	1730
Total	200	4779

**Table 2:** Genes in the Biological Process GO category ‘embryonic organ development’ (GO:0048568). The expression profile of this GO category is affected by the interaction between *FLT3* status and expression-based prognostic score (see Fig. 7). Genes in the also significant offspring term ‘notochord development’ (GO:0030903) are written in bold.

Symbol	Name
MYST3	MYST histone acetyltransferase (monocytic leukemia) 3
SH2B3	SH2B adaptor protein 3
MED1	mediator complex subunit 1
RARG	retinoic acid receptor, gamma
TINAG	tubulointerstitial nephritis antigen
ROR2	receptor tyrosine kinase-like orphan receptor 2
FOXF1	forkhead box F1
FOXF2	forkhead box F2
FOXL2	forkhead box L2
SHH	sonic hedgehog homolog (Drosophila)
SHOX2	short stature homeobox 2
MLL	myeloid/lymphoid or mixed-lineage leukemia (trithorax homolog, Drosophila)
OVOL2	ovo-like 2 (Drosophila)
GLI3	GLI-Kruppel family member GLI3 (Greig cephalopolysyndactyly syndrome)
**GLI1**	glioma-associated oncogene homolog 1 (zinc finger protein)
**GLI2**	GLI-Kruppel family member GLI2
**NOG**	noggin
